# Editorial: The polysaccharides from marine organisms and fungi: Biological functions and molecular mechanisms

**DOI:** 10.3389/fphar.2022.1085346

**Published:** 2022-11-18

**Authors:** Leonel Pereira, Chia-Chuan Chang, Jianxun Ding, Tung-Yi Lin, Zhong Liu

**Affiliations:** ^1^ ARNET Associate Laboratory, Department of Life Sciences, MARE—Marine and Environmental Sciences Centre, University of Coimbra, Coimbra, Portugal; ^2^ Department of Pharmacy, School of Pharmacy, National Taiwan University Taipei, Taipei, Taiwan; ^3^ Changchun Institute of Applied Chemistry, Chinese Academy of Sciences (CAS), Changchun, China; ^4^ School of Medicine, National Yang Ming Chiao Tung University Taipei, Taipei, Taiwan; ^5^ College of Life Science and Technology, Jinan University Guangzhou, Guangzhou, China

**Keywords:** polyporus polysaccharide, iron oxide nanoparticles, medicinal mushrooms, porphyran, antimicrobial peptides

In recent years, there has been an increase in studies on applications of marine algae (macro- and micro-), fungi and other marine organisms, as a result of the identification of different substances synthesized by these organisms. The immense biodiversity and consequent variability in the biochemical composition of the biomass obtained from algae cultures, combined with their genetic improvement and the development of large-scale cultivation technology, have made them a target of interest for the industry, namely the food industry, and pharmaceutical.

Algae and fungi are a potential source of obtaining various biologically active ingredients, such as carotenoids, fatty acids, vitamins, polysaccharides, among others, with an efficiency superior to that verified by traditional terrestrial vegetable cultures, these can be used in the development of functional foods, which has led to the recent increase in commercial interest in marine algae and fungi.

The natural properties of algae allow the extraction of compost with active antimicrobial, antitumoral, antiviral, etc., compounds that are difficult to synthesize, thus allowing greater efficiency in drug development. For In addition, there are currently several cosmetics on the market, aimed at skin care and sun protection, which contain marine algae or fungi extracts in their composition.

In this Research Topic, four original research articles and one review article were published, the latter entitled “Fungal Mushrooms: A Natural Compound with Therapeutic Applications,” (Chugh et al.) in which approximately 130 medicinal activities such as antitumor, immunomodulation, antioxidant, radical scavenging, cardioprotective and antiviral actions are assumed to be produced by the various varieties of medicinal mushrooms.

Of the original research articles, one was published with the title “Polyporus Polysaccharide Ameliorates Bleomycin-Induced Pulmonary Fibrosis by Suppressing Myofibroblast Differentiation *via* TGF-β/Smad2/3 Pathway” (Jiang et al.) and, in this work, it was demonstrated that the polysaccharide Polyporus (PPS) markedly improves bleomycin-induced pulmonary fibrosis in mice.

The authors of the article “Hyaluronic Acid–Stabilized Fe_3_O_4_ Nanoparticles for Promoting *In Vivo* Magnetic Resonance Imaging of Tumors,” (Zhang et al.) reported the creation of hyaluronic acid (HA)-stabilized Fe_3_O_4_ nanoparticles prepared by a hydrothermal coprecipitation method and followed by electrostatic adsorption of HA onto the nanoparticle surface, demonstrating that nanoparticles can be used as effective contrast agents for magnetic resonance imaging (MRI) both *in vitro* in HeLa cells and *in vivo* in a rodents xenografted HeLa tumor model.

The article titled “Porphyran from *Porphyra haitanensis* Alleviates Obesity by Reducing Lipid Accumulation and Modulating gut Microbiota Homeostasis” (Wang et al.). In this article it is mentioned that the polysaccharide porphyran possesses various activities, while the effects of the porphyran from *Neoporphyra haitanensis* (formerly *Porphyra haitanensis*) (Rhodophyta) on obesity are rarely reported. In summary, this study illustrated that porphyran extracted from *N. haitanensis* has the potential to be developed as an anti-obesity agent.

Last but not least, the article entitled “Recombinant Phage Displaying ToAP2D Peptide with Antifungal Activity against *Sporothrix globosa*” (Yan et al.) addresses the antifungal effect on *Sporothrix* and the corresponding mechanism. The authors of this study believe that the recombinant phage inhibits the growth of *Sporothrix* by adjusting the immune response of the mice, inducing *Sporothrix* apoptosis and improving the animal’s survival. Therefore, this study offers a new approach for the preparation of antimicrobial peptides.

